# Prognostic Value of Multiple Draining Lymph Node Basins in Melanoma: A Matched-Pair Analysis Based on the John Wayne Cancer Institute Experience

**DOI:** 10.3389/fonc.2017.00172

**Published:** 2017-08-14

**Authors:** J. Harrison Howard, Junko J. Ozao-Choy, Jason M. Hiles, Myung-Shin Sim, Mark B. Faries

**Affiliations:** ^1^Department of Surgical Oncology, John Wayne Cancer Institute at Saint John’s Health Center, Santa Monica, CA, United States; ^2^Division of Surgical Oncology, The Ohio State University, Columbus, OH, United States; ^3^Department of Biostatistics, John Wayne Cancer Institute at Saint John’s Health Center, Santa Monica, CA, United States

**Keywords:** melanoma, multiple lymph node basin, sentinel lymph node biopsy, prognosis, survival rate

## Abstract

**Background:**

The prognostic significance of multiple draining basins is controversial in melanoma because analyses have not adequately controlled for standard prognostic variables. We hypothesized that an analysis based on prognostically matched pairs of patients with multiple versus single drainage basins would clarify any independent role of basin number.

**Study design:**

We identified patients in our 40-year prospective database, who underwent preoperative lymphoscintigraphy, intraoperative sentinel node biopsy and wide local excision for cutaneous melanoma. Overall survival (OS), disease-specific survival (DSS), and disease-free survival (DFS) were compared in patients with multiple versus single drainage basins after matching by age, sex, Breslow depth, primary site, and stage at diagnosis.

**Results:**

We identified 274 patients with multibasin drainage and 1,413 patients with single draining lymph node basins. Matching yielded 259 pairs (226 trunk, 27 head/neck, 6 extremity). Among matched pairs, multibasin drainage did not affect rates of lymph node metastasis (*p* = 0.84), OS (*p* = 0.23), DSS (*p* = 0.53), overall recurrence (*p* = 0.65), locoregional recurrence (*p* = 0.58), or distant recurrence (*p* = 1.0). Multivariable analysis linked higher T stage, ulceration, older age, and lymph node positivity to decreased DSS (*p* < 0.01) and DFS (*p* < 0.001). Number of drainage basins was not significant on univariable or multivariable analysis.

**Conclusion:**

This analysis, the first to match for standard prognostic factors, suggests that multiplebasin drainage as identified by lymphoscintigraphy has no independent biological or prognostic significance in primary cutaneous melanoma.

## Introduction

Since the advent of lymphoscintigraphy for cutaneous melanoma it has become clear that some primary lesions will drain to more than one lymph node basin (1, 2). Reported rates of multibasin drainage (MBD) are 17–31% and vary related to the location of the primary lesion (3–6). Cutaneous melanomas of the truncal region are most likely to have MBD followed by lesions of the head and neck, and rarely extremity lesions (3). Although sentinel lymph node (SLN) biopsy is recommended for each basin identified during lymphatic mapping (7), the prognostic significance of MBD is unclear.

In the multi-institutional randomized Sunbelt Melanoma trial (3), 351 patients with MBD did not have worse outcomes; in fact their rate of locoregional recurrence was lower than that of the 1,709 patients with single draining lymph node basins. However, a more recent series reported a higher rate of locoregional recurrence, shorter disease-free survival (DFS), and worse melanoma-specific survival in 1,400 patients with MBD (6). Results of smaller studies also are conflicting (4, 5, 8, 9).

Our 1997 report showed that survival was worse in 120 patients with dual-basin metastasis than in 124 patients with the same melanoma burden confined to a single basin (7). Dual-basin metastasis was associated with palpable nodes in 75% of first dissected basins and 43% of second dissected basins. Additionally, the number of tumor-involved nodes was higher in patients with dual-basin metastasis than in those with single basin metastasis. However, this study predated the widespread use of lymphoscintigraphy or SLN biopsy and all patients received regional lymph node dissections. In an effort to add to the current literature regarding the prognostic significance of MBD found with lymphatic mapping of cutaneous melanoma, we have reviewed our experience with this clinical scenario in the modern era. Unlike other retrospective studies, we used a matched-pair analysis to minimize bias in evaluating the true clinical significance of MBD in cutaneous melanoma.

## Materials and Methods

The melanoma database at the John Wayne Cancer Institute (JWCI) has been prospectively maintained for 40 years. Patients have signed written informed consent giving permission to collect tissue, blood, and clinical data for this database. Approval from the JWCI institutional review board was obtained for the current retrospective review and waived the need for additional informed consent for this study. We queried the database to identify all patients who underwent preoperative lymphoscintigraphy and sentinel node biopsy (SNB) for clinically localized melanoma. The study period was January 1991 to December 2010.

### Preoperative Lymphoscintigraphy and Intraoperative Lymphatic Mapping

We have previously reported the technical details of lymphoscintigraphy and SNB at the JWCI (10, 11). However, some details have changed slightly over time due to technological and pharmacologic improvements (12). Lymphoscintigraphy is performed approximately 2–4 h prior to the operative procedure. The operating surgeon marks the primary melanoma site (or the site of prior wide excision) and the patient is sent to the department of nuclear medicine, where filtered technetium ^99m^Tc sulfur colloid (0.5 mCi) is injected into the skin around the marked lesion. Sequential images from a scintillation camera are used to identify the sentinel lymph nodes and the sites are marked on the overlying skin.

The surgical procedure begins with intradermal injection of approximately 1 cc of 1% isosulfan blue dye (Lymphazurin) around the cutaneous melanoma biopsy site. Sentinel nodes are located intraoperatively with a gamma probe as well as by identifying and following the blue-stained lymphatic channels. Maximum *in vivo* and *ex vivo* radioactive counts of the sentinel node(s) and post biopsy basin counts are evaluated and recorded to ensure that all sentinel nodes have been excised. Sentinel nodes are sent to the department of pathology for evaluation of paraffin-fixed sections.

Our study included patients whose primary melanomas drained into basins in the cervical, supraclavicular, axillary, and inguinal chains. Supraclavicular nodes were only included as a separate lymph node basin for patients with melanomas of the upper back, trunk, and shoulder. It has been shown that 6–15% of melanomas in this area can have separate and distinct drainage patterns to both the axillary and/or supraclavicular nodes (13, 14). For head and neck melanomas, supraclavicular nodes were considered part of the cervical chain and not recorded as a separately draining lymph node basin. Epitrochlear and popliteal nodes were not considered separate basins from ipsilateral axillary and inguinal lymph node basins, respectively.

### Matching and Statistical Analysis

Patients with incomplete data on age, sex, Breslow depth, T stage, primary site, nodal status, and patients with mucosal melanomas were excluded from the study. The remaining patients were categorized into those who had single versus multiple draining lymph node basins identified on the lymphoscintigram and during definitive operation. These patients were matched 1:1 by sex, anatomic site of primary melanoma, Breslow depth, age, and stage at diagnosis. Groups were compared before and after matching using the Chi-square test and ANOVA to determine if matching had been successful. Logistic regression was used to evaluate the significance of multiple lymph node basins, site of primary melanoma, Breslow depth, ulceration, and patient age as indicators of lymph node metastasis in the matched groups.

Our primary end points were overall survival (OS), disease-specific survival (DSS), and DFS. Secondary endpoints included total recurrence, locoregional recurrence, and distant recurrence rates. Demographic and prognostic variables were compared by univariable and multivariable analyses using logistic regression to identify factors that may contribute to worse prognosis. Survival curves were constructed using the Kaplan–Meier log-rank test. Significance was set at *p* < 0.05.

## Results

Review of our database revealed 274 patients with multiple draining lymph node basins and 1,413 patients with single draining lymph node basins as identified by lymphoscintigraphy. Matching of these two groups yielded 259 pairs. Comparison of the matched pairs revealed that patient and tumor characteristics were similar for all matching statistics (*p* ≥ 0.84, Table 1).

**Table 1:** Patient demographics.

	Matched		
	One basin	Multiple basins	*p* Value
**Patients**	259	259	
**Sex**			0.92
Male	192	191	
Female	67	68	
**Stage (presentation)**			0.922
I/II	188	187	
III	71	72	
**T stage**			0.989
Unknown	8	7	
T1	101	101	
T2	83	83	
T3	50	49	
T4	17	19	
**Site of primary**			1.0
Extremity	6	6	
Head/neck	27	27	
Trunk	226	226	
**Age**			0.949
0–9	0	1	
10–19	5	5	
20–29	15	17	
30–39	35	39	
40–49	58	52	
50–59	65	58	
60–69	47	51	
70–79	22	26	
80+	12	10	
**Sentinel lymph node**			*p* = 0.8402
Positive	65 (25.1%)	67 (25.9%)	
Negative	194 (74.9%)	192 (74.1%)	

Matched single basin drainage (SBD) and MBD groups each had 259 patients with 226 truncal lesions, 27 head and neck lesions, and 6 extremity lesions. Mean follow-up for matched groups was 80.9 months. All patients in the MBD group had two or three draining basins (Table 2). MBD basins were located bilaterally in 186 patients (67.6%) and unilaterally in 89 patients (32.4%). SLN positivity was 25% (*n* = 65) in the single basin group and 26% (*n* = 67) in the multiple basin group (*p* = 0.84). Of the patients with MBD and SLN metastasis, 45 patients had a positive SLN in only one of the two basins, 16 had positive SLN in the two separate basins, 1 patient had positive SLN in 2 of the 3 basins, and 5 patients had a positive SLN in 1 of the 3 basins (Table 2). There was no difference in the mean number of positive nodes for patients with single basin (1.303) versus multiple basins (1.397) drainage (*p* = 0.5).

**Table 2:** Sentinel lymph node (SLN) status for each individual draining basin in 259 matched patients with multiple draining lymph node basins.

Basins mapped	Positive SLN	*N*	Frequency (%)
2	0 basins	181	69.9
	1 basin	45	17.4
	2 basins	16	6.18
3	0 basins	11	4.25
	1 basin	5	1.93
	2 basins	1	0.39
	3 basins	0	0.0

Total		259	100

**Figure 1:**
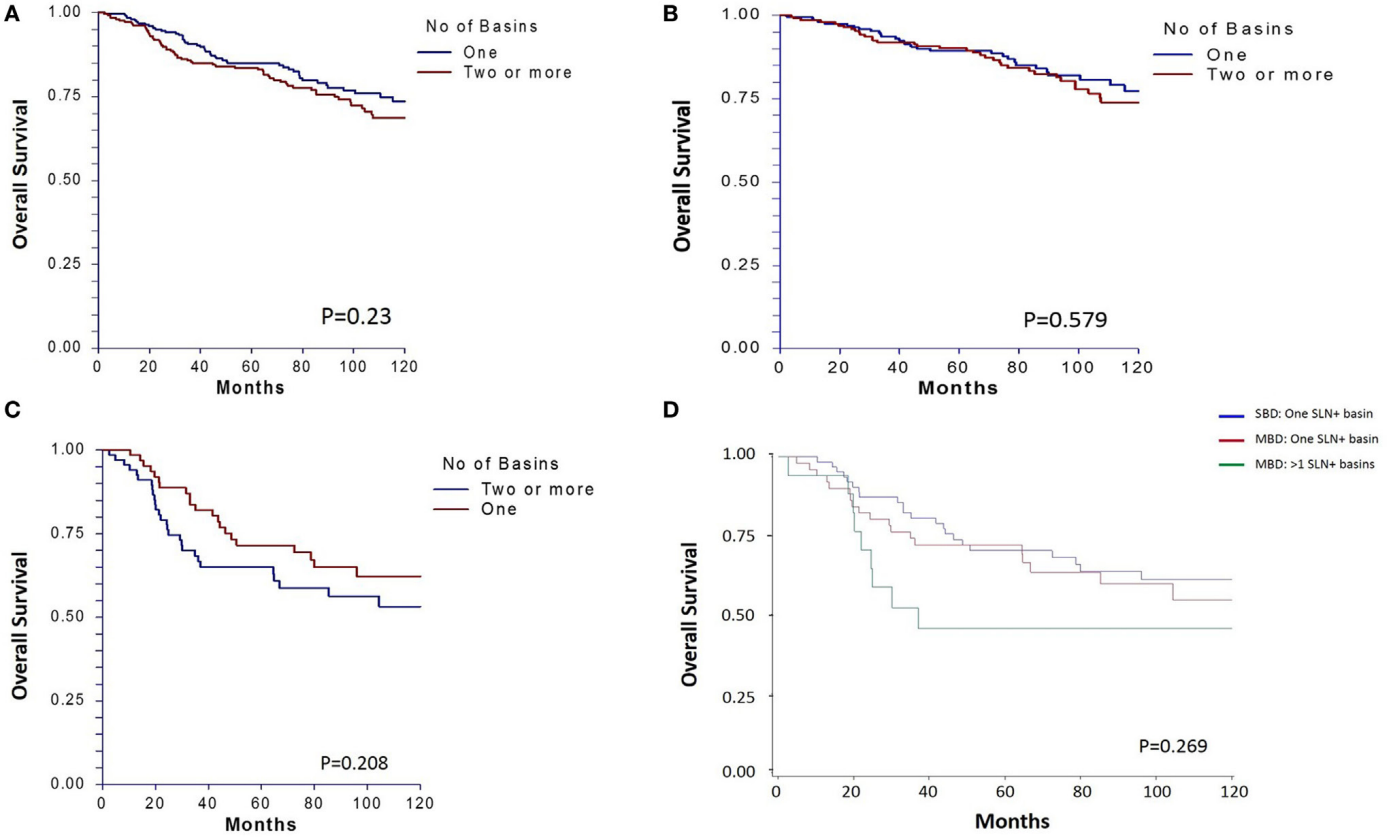
**(A)** Ten-year overall survival for matched patients who had single basin drainage (SBD) and multibasin drainage (MBD). Survival is also shown for **(B)** matched subgroups with tumor-negative sentinel lymph nodes (SLNs) and **(C)** tumor-positive SLNs. **(D)** Survival for SLN-positive patients is further categorized by single basin versus multibasin metastases.

**Figure 2:**
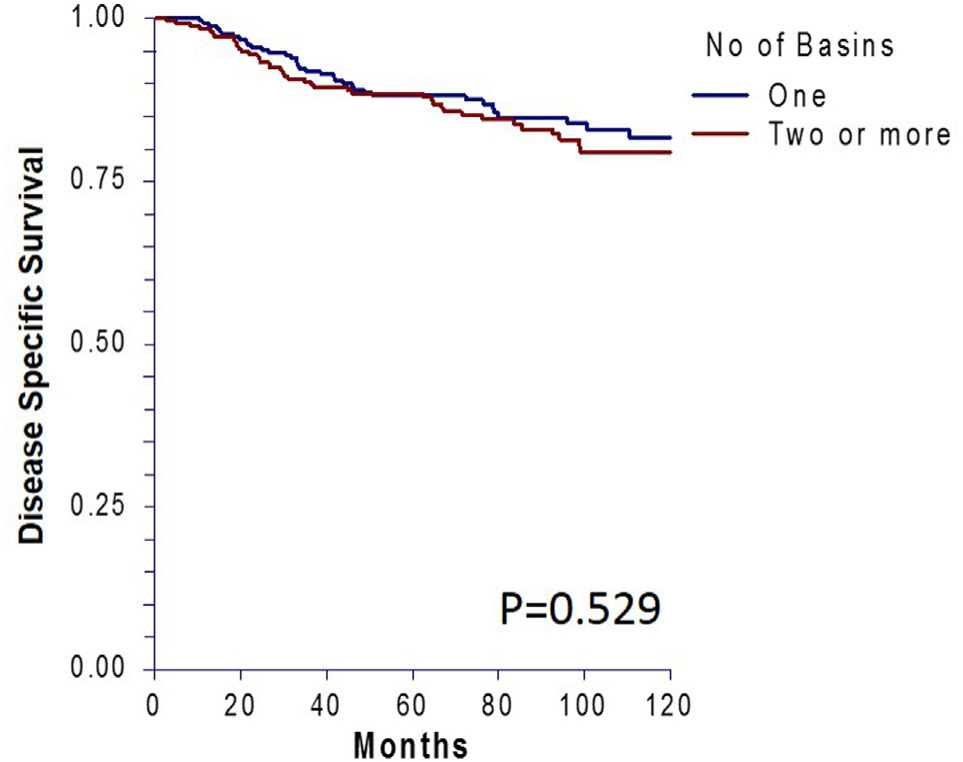
Ten-year disease-specific survival for matched patients who had single versus multiple draining lymph node basins.

There was no significant difference in 10-year OS between the matched groups (*p* = 0.23, Figure 1A). Ten-year OS was 80.3% (CI: 0.75–0.85) for the single basin group compared to 75.7% (CI 0.70–0.81) for the multiple basin group. There was no difference in 10-year OS between the two groups that were either SLN negative (*p* = 0.579, Figure 1B) or SLN positive (*p* = 0.208, Figure 1C). SLN-positive patients showed no difference in 10-year OS whether they had positive nodes in single or multiple basins (Figure 1D). Similarly, DSS also showed no difference between the matched groups (*p* = 0.529, Figure 2). Ten-year DSS was 86.1% for the single basin group and 84.2% for the MBD group. Factors that were predictive of 10-year OS on multivariable analysis between the matched groups included Breslow depth, ulceration, positive SLN, and age (*p* ≤ 0.008 for all variables). Multiple draining lymph node basins did not reach significance as a predictor of OS on multivariable analysis (*p* = 0.129, Table 3).

**Table 3:** Univariable and multivariable analyses of predictors of 10-year overall survival for matched cohorts.

	Univariable	Multivariable
	
	HR (95% CI) *p* Value	HR (95% CI) *p* Value
Draining basins^a^	0.82 (0.57–1.18)	0.75 (0.51–1.09)
One versus multi-basin	0.283	0.129
T stage^a^		
T1: 0.01–1.00	0.10 (0.05–0.21)	0.17 (0.08–0.36)
T2: 1.00–2.00	0.42 (0.25–0.72)	0.50 (0.29–0.86)
T3: 2.01–4.00	0.70 (0.40–1.22)	0.74 (0.42–1.29)
T4: >4.00 (ref)	<0.0001	<0.0001
Ulceration^a^		
Yes versus no	3.56 (2.40–5.28)	1.86 (1.22–1.83)
Unknown versus no	2.95 (1.59–5.48) <0.0001	1.57 (0.80–3.09) 0.015
Age^a^	1.04 (1.02–1.05)	1.04 (1.02–1.05)
	<0.0001	<0.0001
Sentinel lymph node (SLN) positive^a^	2.57 (1.78–3.72)	2.05 (1.38–3.04)
	<0.0001	0.0004
Site^a^		NS
Extremity	0.40 (0.06–2.86)	
Head/neck	1.64 (0.99–2.71)	
Trunk (ref)	0.097^	
Sex^a^	1.12 (0.73–1.71)	NS
Male versus female	0.61	
Stage III versus I/II	3.11 (2.15–4.48)	–
	<0.0001	

*^a^Number of draining basins, T stage, ulceration, age, SLN positive, site, and sex are included in the stepwise model selection for multivariate analysis. Stage was not included in the stepwise model selection and therefore multivariable analysis is not applicable*.

**Figure 3:**
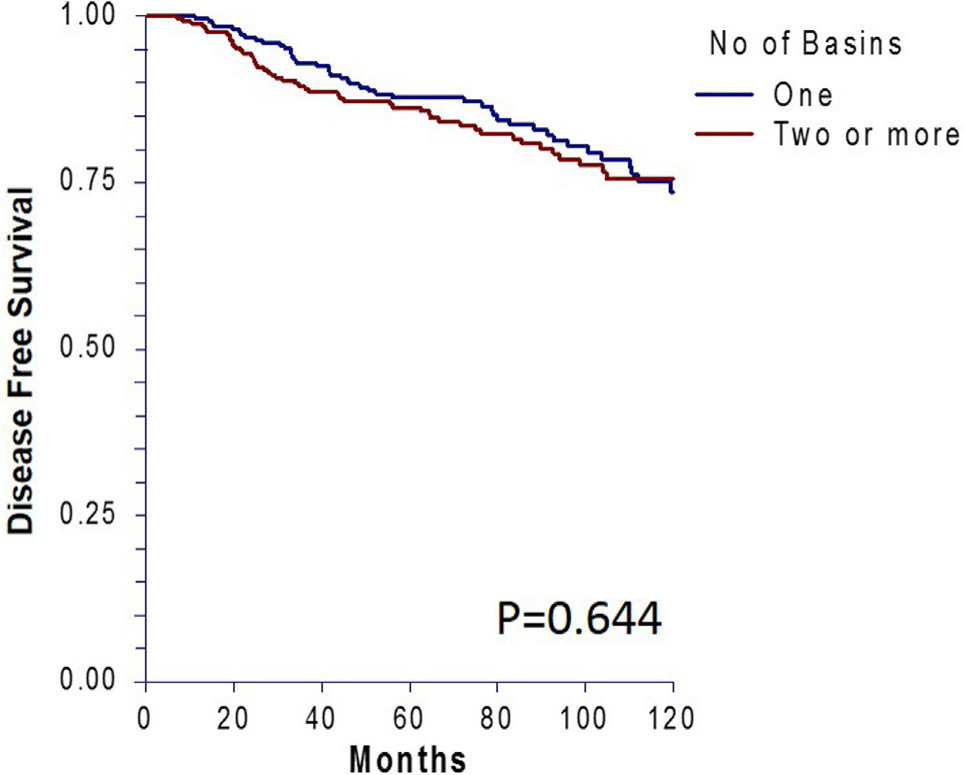
Ten-year disease-free survival for patients who had single versus multiple draining lymph node basins and were matched by standard prognostic factors.

Ten-year DFS revealed no difference between the matched groups (*p* = 0.64, Figure 3). Forty-four patients (17%) in the single basin group and 48 patients (18.5%) in the multiple basin group developed locoregional or distant recurrence (*p* = 0.65). There were 28 locoregional (10.8%) recurrences for the SBD group and 32 (12.4%) for the MBD group (*p* = 0.58). Both groups had 16 patients (6.2%) with distant recurrence (*p* = 1.0, Table 4). When evaluated by SLN status, there was no difference in recurrence rates for patients with a positive node in single (30.8%) or multiple (35.8%) draining basins (*p* = 0.54). This was also true for locoregional recurrences (*p* = 0.25) and distant recurrences (*p* = 0.53).

**Table 4:** Comparison of 10-year overall survival (OS), disease-specific survival (DSS), disease-free survival (DFS), sentinel lymph node (SLN) positivity, and recurrence rates between matched patients with single versus multiple draining lymph node basins.

	Single	Multiple	*p*-Value
10-year OS	80.3%	75.7%	0.23
10-year DSS	86.1%	84.2%	0.53
10-year DFS	83.0%	81.5%	0.64
SLN+	65 (25.1%)	67 (25.9%)	0.84
Recurrence (total)	20 (30.8%)	24 (35.8%)	0.54
Locoregional	12 (18.5%)	18 (26.9%)	0.25
Distant	8 (12.3%)	6 (9.0%)	0.53
Recurrence (total)	44 (17.0%)	48 (18.5%)	0.65
Locoregional	28 (10.8%)	32 (12.4%)	0.58
Distant	16 (6.2%)	16 (6.2%)	1.0

## Discussion

Although other reports have shown that number of drainage basins from primary cutaneous melanoma does not affect survival (3, 4), ours is the first study to match MBD and SBD groups by patient-related and tumor-related prognostic variables. The importance of prognostic matching is demonstrated by critical evaluation of studies that reported a survival impact of MBD but did not control for standard prognostic characteristics (15). For example, one published study found MBD to have an adverse impact on survival, independent of SLN positivity (8). However, the 76 MBD patients had a significantly higher percentage of males and a non-significantly higher rate of thick (>4 mm) primaries than the 190 SBD patients. These imbalances might have contributed to the worse survival in the MBD group (16, 17). Similarly, another study linked MBD to a higher risk of lymph node metastases, but average Breslow thickness was non-significantly greater in MBD patients than SBD patients (5). It is known that melanomas located in different locations may have different behavior. Specifically head and neck melanoma have been shown to have poor survival and truncal melanomas are more likely to have positive sentinel nodes (15, 18). This is particularly important to this study as melanomas in these areas are the most likely to have MDB. By matching, we are able to compare patients with melanomas from the same anatomic areas and avoid comparing tumors from two different locations that may inherently have a different biology that could confound our results.

The data from the current study support no difference in survival for patients with SDB compared to MDB when matched by standard histopathologic prognostic factors. Ten-year OS curves for matched SLN-negative patients are very similar (Figure 1B). There is also no difference in 10-year OS for patients with single versus multiple draining basins that were sentinel node positive. However, while not statistically significant these curves do begin to show some separation at 10 years (Figure 1C), suggesting that perhaps a subset of MDB patients with SLN metastasis may have worse survival—we believe it is likely that those with metastases to be multiple basins (19). When 10-year OS is evaluated by the number of basins with a positive SLN (Figure 1D), the curves between patients with a single positive basin and multiple positive basins appear distinct, suggesting that patients with positive SLN in multiple basins may represent a subgroup of MDB patients with worse 10-year OS. While not reaching significance in the current study, this hypothesis is consistent with our previous study of patients with metastases in multiple basins, which concluded that the same total tumor burden of the regional nodes will carry a worse prognosis if it involves more than one lymphatic drainage basin (7). While having MBD on lymphoscintigraphy may not be a poor prognostic finding, it has been shown to be prognostically worse to have either microscopic or palpable positive nodes in multiple basins as compared to the same number of positive nodes in a single basin (7, 19). Thus having metastases in multiple basins may be the true indicator of poor prognosis, not simply finding drainage to multiple basins on lymphoscintigraphy. The nearly identical survival curves for patients with negative sentinel nodes (Figure 1B) and for patients with positive nodes in only one basin (Figure 1D) support this theory.

Most studies have shown that MBD does not correlate with SLN metastasis (3, 4, 8). The clinical finding with the most variability among studies is the rate of recurrence for patients with MBD. Several institutions have reported increased recurrence rates for patients with multiple basins (6, 8, 9), while one showed worse recurrence rates for patients with a single draining basin (3). We found that number of drainage basins did not affect DFS, overall recurrence, locoregional, or distant recurrence, regardless of SLN status.

Whether the phenomenon of MBD is purely based on anatomical location of the primary tumor or influenced by tumor biology remains unknown. Biologic factors that could theoretically increase the likelihood of multibasin drainage include ulceration, regression, lymphatic clogging, or possibly “collateral route” spread as a result of lymphangiogenesis induced by the primary tumor (8). Additionally, it has been suggested that tumors with MBD may be predisposed to distant, blood-borne metastases (8). Our findings do not rule out these hypotheses but do demonstrate that once appropriately controlled, the rate of developing distant metastases was identical between the two groups.

Despite our long follow-up and best efforts to minimize bias, the limitations of a single-institution retrospective study remain. Pathological findings including mitoses, ulceration, and regression were not routinely reported during our study period and were therefore not used to correlate survival or recurrence for the two groups. We are unable to comment on the independent significance of MBD on SLN metastasis in our matched groups since stage at presentation (which integrates the results of SNB) was one of the matching criteria and thus both groups had the same number of patients with regional microscopic metastases at presentation. By using stage at presentation as a matching criterion, we could compare long-term survival and recurrence data for two large groups of very similar patients. Had patients not been matched by stage, a disproportionate distribution of stage I/II and stage III cases could have biased survival and recurrence data. While multivariable analysis, and not matching, may be the standard method of retrospectively evaluating two groups to correct for confounders and population heterogeneity, all previous studies of MBD have used these methods with conflicting results (3–6, 8, 9, 20). Given the inconsistent conclusions found with multivariable analysis, we hope to bring a new perspective to this clinical scenario by using the alternative statistical method of base pair matching to add to the understanding of the true prognostic value of MBD when found on lymphoscintigraphy.

Unlike several other studies, our data show no difference in OS or DFS of patients whose primary cutaneous melanoma drains to single versus multiple nodal basins (5, 6, 8, 9). This is the first study to evaluate this clinical scenario in a matched-pair analysis. MDB may only be a factor for 10-year OS for the small group of patients who have multiple SLN-positive basins compared to patients with the same disease burden in a single basin but our study is not powered to definitively make these conclusions. Our data suggests that the clinical finding of MDB on lymphoscintigraphy does not necessarily portend a worse prognosis when compared to patients with similar primary tumors and only one draining basin. When patients have multiple draining lymph node basins from a primary cutaneous melanoma, each basin should be treated according to the standard of care and physicians can appropriately counsel patients based on the findings of SNB, primary tumor characteristics, and patient demographics in regard to treatment, surveillance, and prognosis.

## Author Note

Presented in part at the American College of Surgeons Clinical Congress Scientific Paper Sessions, Chicago, IL, September 30–October 4, 2012.

## Author Contributions

Authors were involved in writing manuscript (JH, JO-C, M-SS, and MF), critical review (JH, MF, JH, JO-C, and M-SS), data collection (JH, JO-C, JH, and M-SS), analysis and interpretation of data (JH, JO-C, M-SS, and MF), and statistical analysis (M-SS, JH, and MF).

## Conflict of Interest Statement

The authors declare that the research was conducted in the absence of any commercial or financial relationships that could be construed as a potential conflict of interest.
